# Reciprocal Regulation of V-ATPase and Glycolytic Pathway Elements in Health and Disease

**DOI:** 10.3389/fphys.2019.00127

**Published:** 2019-02-15

**Authors:** Summer R. Hayek, Hallie S. Rane, Karlett J. Parra

**Affiliations:** Department of Biochemistry and Molecular Biology, University of New Mexico Health Sciences Center, Albuquerque, NM, United States

**Keywords:** V-ATPase, glycolysis, glucose, metabolism, TORC1, yeast, human, cancer

## Abstract

The ability of cells to adapt to fluctuations in glucose availability is crucial for their survival and involves the vacuolar proton-translocating ATPase (V-ATPase), a proton pump found in all eukaryotes. V-ATPase hydrolyzes ATP via its V_1_ domain and uses the energy released to transport protons across membranes via its V_o_ domain. This activity is critical for pH homeostasis and generation of a membrane potential that drives cellular metabolism. A number of stimuli have been reported to alter V-ATPase assembly in yeast and higher eukaryotes. Glucose flux is one of the strongest and best-characterized regulators of V-ATPase; this review highlights current models explaining how glycolysis and V-ATPase are coordinated in both the *Saccharomyces cerevisiae* model fungus and in mammalian systems. Glucose-dependent assembly and trafficking of V-ATPase, V-ATPase-dependent modulations in glycolysis, and the recent discovery that glucose signaling through V-ATPase acts as a molecular switch to dictate anabolic versus catabolic metabolism are discussed. Notably, metabolic plasticity and altered glycolytic flux are critical drivers of numerous human pathologies, and the expression and activity of V-ATPase is often altered in disease states or can be pharmacologically manipulated as treatment. This overview will specifically discuss connections between V-ATPase and glycolysis in cancer.

## Introduction

**FIGURE 1:**
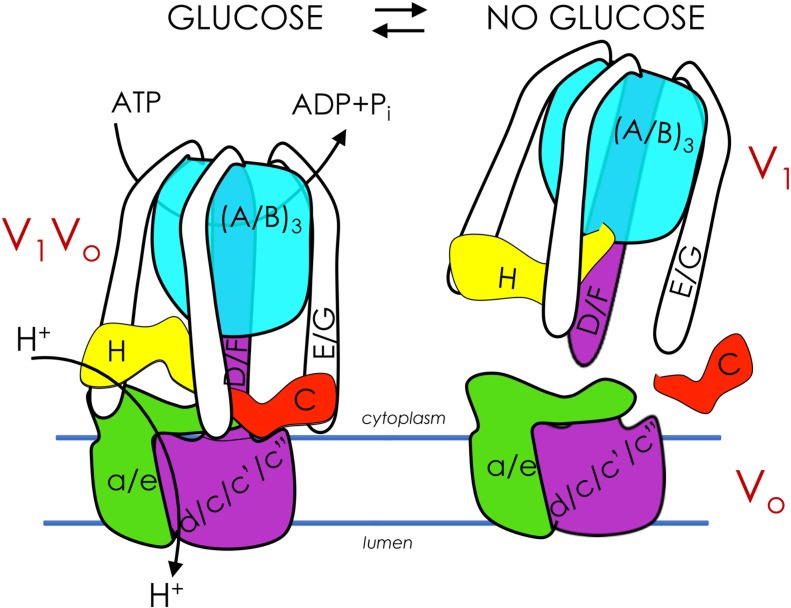
Glucose regulates assembly of V-ATPase. V-ATPases are members of the rotary ATPase protein family. V-ATPase has two domains, V_1_ (peripheral, subunits A-H) and V_o_ (membrane, subunits a, c, c’, c”, d, e) that are highly conserved. V_1_V_o_ couples ATP hydrolysis to active transport of protons across membranes, generating an acidic pH in lysosomes/vacuoles, endosomes, and the Golgi (*left*). V_1_V_o_ separate from each other after glucose depletion, inhibiting V-ATPase function (*right*). V-ATPase reassembly is triggered by glucose readdition and is intertwined with glycolysis.

Vacuolar proton-translocating ATPase (V-ATPase) is a highly efficient energy conversion machine and a member of the rotary ATPase protein family. V-ATPase couples ATP hydrolysis to active proton transport across membranes. V-ATPase acidifies lysosomes/vacuoles, Golgi, and endosomes of all eukaryotes and can be recruited to the plasma membrane of certain specialized mammalian cells to aid in proton export from the cell (Cotter et al., 2015). In intracellular compartments, V-ATPase is critical for a plethora of cellular processes, including protein processing and secretion, endocytosis and vesicle trafficking, zymogen activation, and autophagy (Forgac, 2007; Cotter et al., 2015). V-ATPase is comprised of a peripheral, cytoplasmic V_1_ domain (V_1_ subunits A through H) attached to a membrane-embedded V_o_ domain (V_o_ subunits a, c, c’, c", d, e (Figure 1, *left*). The V_1_ domain binds cytosolic ATP while the V_o_ domain binds cytosolic protons. When ATP is hydrolyzed in the V_1_ domain, the energy released drives relative rotation of subunits, thus pumping protons and establishing a pH gradient across the membrane that is essential for secondary transport systems (Parra et al., 2014). V-ATPase structure, subunit composition, and mechanism of rotational catalysis are largely conserved from yeast to humans. The difference is that while yeast express functional isoforms of V_o_ subunit a only, mammalian V-ATPase lacks subunit c’ and expresses two to three isoforms of most subunits. Several of these subunit isoforms are cell- and even membrane-specific (Kane, 2006; Cotter et al., 2015). In the different membrane microenvironments, V-ATPase participates in diverse cellular processes; among these are the events intertwining V-ATPase and metabolism described in this review.

**FIGURE 2:**
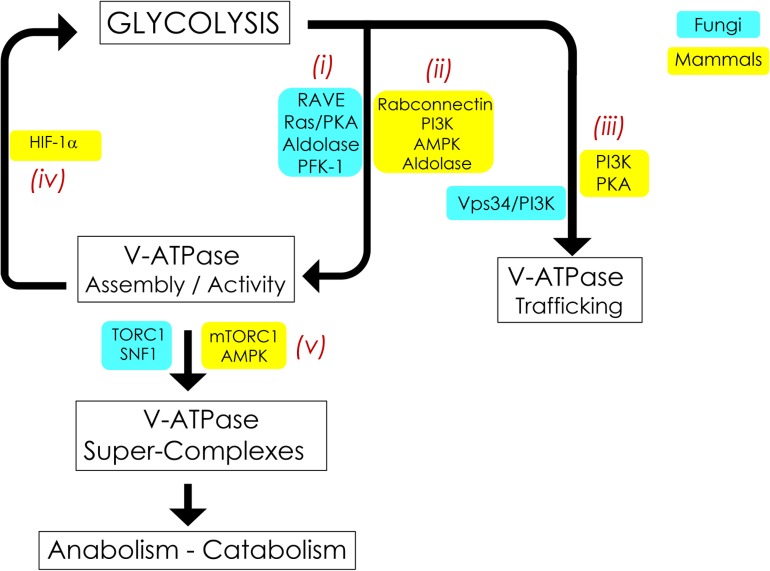
Signaling pathways interconnecting V-ATPase and glycolysis. V-ATPase assembly, activity, and cellular membrane distribution reflect glucose and energy levels within the cell. PKA, AMPK, and PI3K are the common glucose-sensitive signaling pathways that regulate assembly **(i, ii)** and trafficking **(iii)** of V-ATPase in fungi and mammals. Reciprocal regulation of glycolysis by V-ATPase **(iv)** appears to be unique to mammals and is modulated by alterations in HIF-1. V-ATPase is also crucial for metabolic reprogramming **(v)**; this entails assembly of V-ATPase, aldolase, mTORC1, and AMPK into evolutionarily-conserved super-complexes.

Many proliferating cells preferentially use glucose as an energy source, making metabolic plasticity and adaptation to low-glucose environments critical to success. As such, V-ATPase activity is closely associated with glucose and glycolysis in species ranging from the simplest single-celled eukaryotic organism to complex, multicellular mammals. The first reports of glucose-induced V-ATPase regulation were noted over twenty years ago (Kane, 1995; Sumner et al., 1995) and novel insights into the interplay between V-ATPase and glycolysis steadily continue. This review will examine the signaling pathways involved in this process, in both the well-studied *Saccharomyces cerevisiae* model fungus and in mammalian cells (Figure 2). We will discuss reversible disassembly and regulated trafficking of V-ATPase in response to glucose, reciprocal regulation of glycolysis by V-ATPase, and the very recent and intriguing findings that disparate metabolic cues are coordinated in a lysosomal super-complex dependent upon V-ATPase.

Adaptation to changes in glucose concentration is particularly important for cancer cell survival, as nutrients can be limiting, especially during anti-angiogenic therapy (McIntyre and Harris, 2015). Indeed, altered glycolytic flux is a hallmark of cancer: cancer cells use glycolysis (as opposed to oxidative phosphorylation) at much higher rates than non-cancerous cells, even when oxygen concentrations are high (Warburg, 1956). This “Warburg Effect” produces excess lactic acid, and V-ATPase is necessary to remove this acid load from the cytosol (Sennoune and Martinez-Zaguilan, 2012). As such, cancer cells and tumors up-regulate both intracellular and cell-surface V-ATPase (Cotter et al., 2015; Fordyce et al., 2016), and treatment with V-ATPase inhibitors leads to cell death and could be used for improved cancer prognosis (Perez-Sayans et al., 2009). V-ATPase at the cell surface exports protons to acidify the extracellular space, contributing to tumor invasion (McGuire et al., 2016), and V-ATPase expression is noticeably increased in tumors and cells lines that are particularly aggressive, metastatic, and resistant to therapy (Sennoune and Martinez-Zaguilan, 2012; Cotter et al., 2015). This review will highlight the mechanistic pathways underpinning the unique relationship between glycolysis and V-ATPase in cancer.

## Glycolysis Influences Regulated Assembly of V-ATpase

Cells control V-ATPase activity in several ways, from feedback inhibition and disulfide bond formation at the catalytic sites to more sophisticated modifications such as reversible disassembly of the V_1_V_o_ complex (Figure 1, *right*). During disassembly, V_1_ subunit C, which acts as a bridging subunit between the V_1_ and V_o_ domains, leaves the complex (Kane, 1995; Tabke et al., 2014), allowing the remaining V_1_ domain to separate from V_o_. A second V_1_ subunit, subunit H, then undergoes a conformation change that prevents free V_1_ from hydrolyzing ATP, preventing energy depletion in the absence of proton transport (Parra et al., 2000; Cipriano et al., 2008; Diab et al., 2009; Sharma et al., 2018). Upon dissociation, passive proton translocation across free V_o_ is blocked (Couoh-Cardel and Wilkens, 2015). Therefore, disassembly inhibits V-ATPase function *in vivo*. This process is fully reversible upon the readdition of V_1_ subunit C (Kane, 1995). In insect cells, reassembly requires phosphorylation of subunit C (Voss et al., 2007), although it is unclear if this also happens in fungi and humans, and if so, which kinase(s) is involved. Notably, although disassembly involves microtubules (Xu and Forgac, 2001), no other known disassembly factors are involved. Additionally, catalytically active V-ATPase is required for disassembly in both yeast and human cells (Parra and Kane, 1998; Shao and Forgac, 2004; Xie et al., 2004). Reassembly uses a V-ATPase-exclusive chaperone (Smardon et al., 2015). These findings suggest that V-ATPase is naturally prone to disassemble (Stewart and Stock, 2012).

Glucose depletion is the primary trigger for V-ATPase disassembly and was first demonstrated in yeast (Kane, 1995) and insects (Sumner et al., 1995), followed later by mammalian cells (Nakamura, 2004; Sautin et al., 2005). Glucose readdition, which reactivates glycolysis, initiates rapid reassembly in both yeast (Kane, 1995; Parra and Kane, 1998) and mammals (Sautin et al., 2005; Marjuki et al., 2011). This disassembly/reassembly cycle serves a critical function in the cell: V-ATPase disassembly during glucose starvation conserves cellular ATP stores during times of energy stress, and subsequent glucose-induced reassembly and activity helps reduce excess cytosolic acidification stemming from high rates of glycolysis (Parra et al., 2014; Cotter et al., 2015). Studies in yeast have demonstrated that disassembly/reassembly is proportional to glucose concentration and the flux of glycolysis, suggesting that V-ATPase assembly is exquisitely tuned to cellular energy needs (Kane, 1995; Parra and Kane, 1998; Chan and Parra, 2014, 2016). Notably, a recent study in cultured mammalian cells demonstrated for the first time that acute glucose depletion can increase V-ATPase assembly and activity, rather than leading to disassembly as expected (McGuire and Forgac, 2018; Parra and Hayek, 2018). Future studies will be necessary to explain the discrepancies between this study and earlier work, although the authors hypothesized that elevated V-ATPase activity and lysosomal acidification are necessary to activate autophagy enzymes during periods of glucose starvation in human cells (Yoshimori et al., 1991; Feng et al., 2014; Mauvezin and Neufeld, 2015). Mammalian V-ATPase may also respond differently to acute and chronic glucose depletion. For example, V-ATPase activity may be required initially (first 10 min after glucose depletion) but is followed by inevitable disassembly during long starvation periods (e.g., 18 h) (Sautin et al., 2005) to diminish the high energy cost of V-ATPase proton pumping.

In *S. cerevisiae*, V-ATPase assembly in response to glucose readdition relies in part on the Regulator of ATPases of Vacuoles and Endosomes (RAVE) (Figure 2i). RAVE is a three-part chaperone consisting of the Skp1p adaptor protein and two functional subunits, Rav1p and Rav2p (Seol et al., 2001; Smardon et al., 2015). RAVE stabilizes free V_1_ in a form that is competent for reassembly (Smardon et al., 2002) and aids in the readdition of V_1_ subunit C back into the assembled V_1_V_o_ complex for full V-ATPase function (Smardon and Kane, 2007). RAVE assembles V-ATPase complexes located specifically in the vacuole by binding to the vacuolar V_o_ subunit a isoform Vph1p. Golgi/endosome complexes, which contain V_o_ subunit a isoform Stv1p, do not practice regulated disassembly (Smardon et al., 2014, 2015). The RAVE assembly pathway may be conserved in humans via the mammalian rabconnectin homologues (Figure 2ii), the best studied of which is rabconnectin-3 (Sethi et al., 2010; Einhorn et al., 2012). Of note, in addition to glucose-induced V-ATPase assembly, RAVE assembles vacuolar V-ATPase biosynthetically in a glucose-independent manner (Smardon et al., 2002). This suggests that, at least in fungi, RAVE is not the central glucose sensor for V-ATPase assembly. We will next examine other glucose-sensitive signaling pathways and glycolytic enzymes that may coordinate glycolytic status and V-ATPase regulated disassembly in yeast and mammals; these pathways are summarized in Figure 2.

### Glucose-Sensitive Signaling Pathways

**FIGURE 3:**
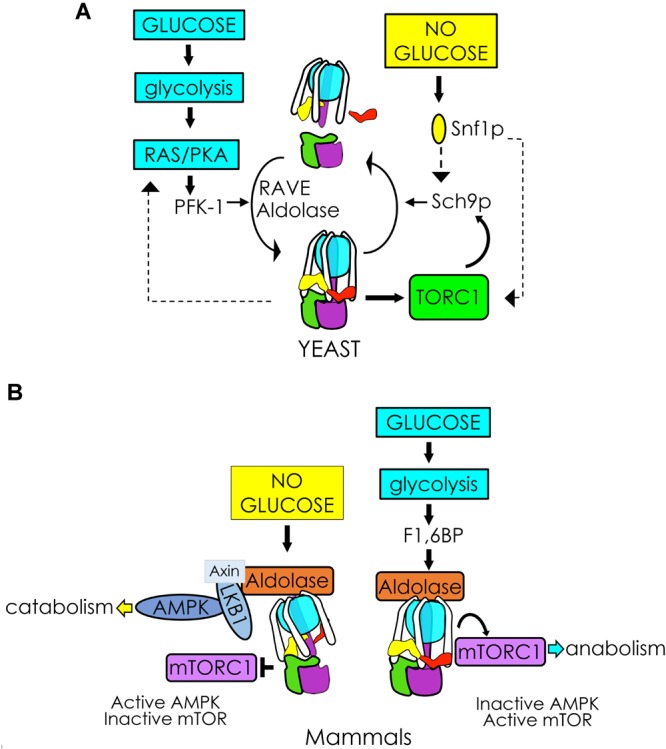
V-ATPase integrates glycolytic signals to control cellular metabolism. **(A)** In yeast, glycolysis activates RAS/PKA and PFK-1, facilitating V_1_V_o_ assembly/activity via RAVE and aldolase. Glucose-induced V-ATPase assembly and PKA activation may function in a positive feedback loop enhancing V_1_V_o_ assembly (*left*). V-ATPase is also necessary for TORC1 phosphorylation of the protein kinase Sch9p, which stimulates metabolism in response to glucose. The V-ATPase-TORC1 interplay is reciprocal. After glucose removal, Snf1p (yeast AMPK) phosphorylates Sch9p, and may participate in a negative feedback response to disassemble V-ATPase (*right*). **(B)** In mammals, glucose removal facilitates assembly of a lysosomal AXIN/LKB1/aldolase protein complex to activate AMPK. Activation of AMPK pulls the V-ATPase-Ragulator complex away from mTORC1 and leads to mTORC1 inactivation (*left*). Glycolytic F1,6BP binds to aldolase, disrupting the lysosomal AMPK activating complex and activating mTORC1 when glucose is available (*right*).

Thus far in *S. cerevisiae*, the best understood glucose-sensitive signaling mechanism controlling V-ATPase assembly is the Ras/cAMP/Protein Kinase A (PKA) pathway (Figure 2i; Bond and Forgac, 2008). Active Ras is a GTP-coupled protein, and Ras activity is negatively regulated by the Ira1p and Ira2p GTPase-activating proteins (GAPs). Glucose addition inhibits Ira1p and Ira2p, and GTP-bound Ras can then activate adenylate cyclase to produce cAMP. Elevated levels of cAMP trigger dissociation of the PKA regulatory subunit to activate the kinase activity of PKA. The downstream effect of glycolytic signaling through PKA is enhanced V-ATPase assembly, although the specific PKA phosphorylation target that triggers complex formation is still unclear. In insect cells, PKA phosphorylates the V_1_ subunit C subunit of V-ATPase (Voss et al., 2007), which is an attractive target, given its essentiality in V-ATPase reassembly. However, a similar phosphorylation event has not been detected in yeast thus far. PKA is also known to phosphorylate and activate 6-phosphofructo-1-kinase (PFK-1) (Ptacek et al., 2005), a glycolytic enzyme that affects V-ATPase assembly (discussed in more detail below), offering the intriguing possibility that seemingly disparate PKA and PFK-1 glucose signaling pathways may be interconnected via V-ATPase (Figure 3A, *left*). Interestingly, there is evidence to suggest that V-ATPase assembly may occur in response to glycolysis-induced cytosolic acidification, leading to changes in PKA (Purwin et al., 1986; Dechant et al., 2010). After glucose depletion, glucose readdition activates PKA, which promotes V-ATPase reassembly. In turn, increased V-ATPase assembly helps sustain cytosolic pH homeostasis and may activate PKA signaling, which upregulates glycolysis, enhances V-ATPase assembly, and mediates the rapid transition from respiratory to fermentative growth. This suggests that glucose-induced V-ATPase assembly and PKA activation function in a positive feed-back loop (Parra et al., 2014).

In mammals, the phosphoinositol-3 kinase (P13K) pathway affects glucose-triggered assembly (Figure 2ii; Nakamura, 2004; Sautin et al., 2005). Chronic glucose depletion triggers increased V-ATPase disassembly, lowered V-ATPase activity, and defective acidification in renal epithelial cell models. After disassembly, V-ATPase requires PI3K activity for reassembly; specifically, these phenotypes can be saved by adding back glucose or a constitutively active PI3K catalytic subunit (Sautin et al., 2005). This appears related to microfilament formation or phosphorylation of lipids to change the lipid environment (Sautin et al., 2005). The first glycolytic intermediate, glucose-6 phosphate (G6P), may be the initial signal for reassembly in kidney cell cultures. The non-metabolizable glucose analog 2-deoxyglucose can substitute for glucose in triggering V_1_V_o_ reassembly, indicating that downstream glycolysis beyond G6P is not necessary (Sautin et al., 2005). This is notably different from yeast, which require metabolism beyond G6P formation and PKA to reassemble V_1_V_o_ (Parra and Kane, 1998; Bond and Forgac, 2008).

As mentioned previously, V-ATPase assembly actually increases during acute glucose depletion in renal cell cultures (McGuire and Forgac, 2018; Parra and Hayek, 2018). This response requires the metabolic sensor AMP-activated protein kinase (AMPK) (Figure 2ii). Interestingly, in the intercalated cells of the kidney collecting ducts, AMPK phosphorylates the V-ATPase catalytic subunit V_1_ subunit A at Ser-384 (Alzamora et al., 2013). Phosphorylation inhibits V-ATPase-dependent proton secretion during glucose depletion and stimulates V-ATPase internalization from the apical plasma membrane to the cytoplasm, apparently without changing V_1_V_o_ association. Both inactivation and redistribution were prevented in a subunit A S384A mutant, despite AMPK activation. Together, these studies argue that V-ATPase is a direct downstream effector of AMPK in kidney.

### Glycolytic Enzymes

The rate-controlling enzyme of glycolysis, PFK-1, interacts with V-ATPase and copurifies with vacuolar membranes (Figure 2i; Su et al., 2003; Chan and Parra, 2014). PFK-1 produces fructose-1,6 bisphosphate (F1,6BP), which couples glycolysis to RAS signaling (Peeters et al., 2017). Moreover, F1,6BP allosterically activates pyruvate kinase, stimulating glycolysis. PFK-1 senses the energy status of the cell: PFK-1 is inactivated by ATP and activated by AMP, thus suppressing and accelerating the flux of glycolysis, respectively. Yeast mutants lacking either of the two PFK-1 structural genes, *PFK1* or *PFK2*, metabolize glucose at 40% reduced rates compared to wild-type cells; these mutants assemble V-ATPase at steady state, but the proton pump is only partially active (Chan and Parra, 2014). Increasing glucose two-fold proportionally increases glycolytic flux in *pfk2Δ* cells. Under these conditions, V-ATPase binding to the remaining PFK-1 subunit (Pfk1p/α) increases and proton transport and acidic vacuolar pH are restored (Chan and Parra, 2016). This suggests that the PFK-1 α-subunit may regulate V-ATPase by fine-tuning proton transport in alignment with the glycolysis flux. Additionally, *pfk2Δ* cells cannot sufficiently reassemble V_1_ and V_o_ after resupplementation with glucose. Glucose-dependent reassembly is 40% reduced in this strain (Chan and Parra, 2014). Furthermore, *pfk2Δ* accumulates substantially high levels of V_1_ - RAVE complexes in the cytoplasm, indicating that PFK-1 passes the glucose signal to RAVE, initiating V_1_- RAVE dissociation and subsequent V_1_V_o_ reassembly. The interaction between PFK-1 and V-ATPase may be biomedically relevant. In cancer cells, V-ATPase and PFK-1 are upregulated and considered important for metabolic reprogramming due to the Warburg effect (Webb et al., 2015; Stransky et al., 2016). In human renal cells, PFK-1 binds the C-terminus of the V-ATPase V_o_ subunit a isoform a4 (V_o_a4-CT); naturally occurring genetic mutations disrupt V_o_a4-CT association with PFK-1 and cause recessive distal renal tubular acidosis (Su et al., 2003, 2008).

The next enzyme in the glycolytic pathway, aldolase, catalyzes the aldol cleavage reaction that converts F1,6BP into dihydroxyacetone phosphate and glyceraldehyde 3-phosphate. Aldolase interacts with V-ATPase and influences V-ATPase function (Figure 2i, Lu et al., 2001, 2007, Zhang et al., 2017). Yeast aldolase may stabilize V_1_V_o_ through binding to V_1_ subunits E and B and V_o_ subunit a (Lu et al., 2004, 2007). Mammalian aldolase (isoforms ALDOA/B/C) also interacts with the V-ATPase V_1_ subunit E directly and coimmunoprecipitates with V-ATPase from bovine kidney microsomes and osteoclasts, further linking glucose metabolism to V-ATPase function (Figure 2ii; Lu et al., 2001). Moreover, V-ATPase and aldolase are components of a lysosomal super-complex crucial for metabolic reprogramming by mTOR and AMPK when glucose is not available (Kemp and Oakhill, 2017; Zhang et al., 2017), as described later in this review (Figure 3B).

## Glycolysis Influences Regulated Trafficking of V-ATpase

In addition to regulating V-ATPase assembly and activity, glucose sensing plays a role in V-ATPase trafficking (Figure 2iii). In yeast, defects in the glucose-sensing enzyme Vps34p (yeast phosphoinositol-3 kinase, or PI3K) lead to defects in V-ATPase activity; this is due primarily to trafficking defects rather than assembly defects (Gary et al., 1998; Sambade et al., 2005; Li and Kane, 2009). In mammalian kidney epithelial cells, V-ATPase movement to the plasma membrane also requires PI3K (Nakamura, 2004; Sautin et al., 2005). Thus, glucose not only increases V_1_V_o_ assembly but also stimulates translocation of assembled V-ATPase pumps from internal membranes to the apical plasma membrane through PI3K signaling.

PKA signaling also regulates trafficking of V-ATPase in mammals. In kidney intercalated cells, accumulation of active V-ATPase proton pumps at the plasma membrane is enhanced by PKA phosphorylation of Ser-175 in V-ATPase subunit A through a cascade of reactions that involves carbonic anhydrase, soluble adenylate cyclase, and cAMP (Pastor-Soler et al., 2008; Alzamora et al., 2010). While PKA-dependent trafficking/activation occurs after a variety of stimuli, including changes in extracellular bicarbonate concentration and pH, it is notable that glucose does not appear to stimulate this process. Interestingly, in fungal V-ATPase, Ser-175 is in a glucose-sensing region of subunit A that is linked to V_1_V_o_ regulated disassembly (Shao and Forgac, 2004). This region, known as the non-homologous region of subunit A, is highly conserved from yeast to humans. In mammals, this conserved region of V_1_ may be functionally divergent, explaining why glucose does not seem to stimulate PKA-dependent V-ATPase trafficking in kidney cells.

## V-ATpase Influences Glycolysis

This review has thus far discussed ways in which glycolysis influences V-ATPase activity. We now turn our attention to the reciprocal pathway, in which alterations to V-ATPase can affect glycolysis, particularly through HIF-1α signaling (Figure 2iv). This alternate signaling pathway has not yet been demonstrated in fungal cells and appears to be unique to mammalian biology.

In human cells, alterations in V-ATPase activity influence downstream glycolysis through the transcription factor Hypoxia Inducible Factor 1 (HIF-1). When molecular oxygen is available, the H1F-1 subunit α (HIF-1α) is hydroxylated by prolyl hydroxylase (PHD), leading to von Hippel-Lindau (VHL) tumor suppressor binding and subsequent ubiquitination and degradation (Aragones et al., 2009; Semenza, 2010). At low oxygen, or when V-ATPase is inhibited, HIF-1α and HIF-1β dimerize for activation (Lim et al., 2006; Zhdanov et al., 2013; Forgac, 2018; Licon-Munoz et al., 2018). Nuclear HIF-1α promotes metabolic adaptations to ensure cell survival, including an upregulation in the expression of genes responsible for glycolytic metabolism. Specifically, HIF-1α increases expression of *GLUT1* and *GLUT3* glucose transporter genes, which leads to a downstream increase in glucose uptake into cells. HIF-1α also increases expression of glycolytic enzymes and lactate dehydrogenase, and decreases citric acid metabolism and oxidative phosphorylation (Zhdanov et al., 2013; Courtnay et al., 2015). This series of events drives the phenomenon known as the Warburg effect, in which cancer cells preferentially use anaerobic glycolysis rather than oxidative phosphorylation, even in the presence of oxygen.

How does V-ATPase inhibition lead to elevated HIF-1 signaling and thus increased glycolysis? Defective endo-lysosomal pH impairs receptor-mediated endocytosis of the transferrin receptor, which leads to cell-wide iron deprivation. Iron plays a role in the HIF-1α degradative process (Miles et al., 2017; Forgac, 2018; Licon-Munoz et al., 2018). Specifically, iron serves as co-factor for PHD (Aragones et al., 2009; Courtnay et al., 2015; Maxwell and Eckardt, 2016; Miles et al., 2017). Cells lacking V-ATPase activity either by genetic disruption (Miles et al., 2017) or pharmacological inhibition (Licon-Munoz et al., 2018) display decreased HIF-1α hydroxylation and turnover and increased HIF-1α translocation to the nucleus. These effects are reversible following iron repletion. Other mechanisms underlying V-ATPase regulation of HIF expression have been described. For example, free V_o_ subunit c binds to the *N*-terminus of HIF-1α to stabilize it and prevent degradation by VHL (Lim et al., 2007). In addition, since V-ATPase regulates cytoplasmic pH, inhibition of V-ATPase leads to proton build-up in the cytosol. Cytosolic acidosis increases HIF-1 expression by inducing nucleic sequestration of VHL, rendering it inactive and allowing HIF-1α to evade degradation (Mekhail et al., 2004).

Remarkably, V-ATPase inhibition promotes pro-survival mechanisms through HIF-1 and yet can also kill cancer cell lines (Forgac, 2018; Licon-Munoz et al., 2018). High rates of glycolysis in cancer cells leads to cytosolic acidosis, which is normally relieved by compensatory up-regulation of V-ATPase proton pumping activity at endo-lysosomal membranes and particularly at the plasma membrane (Forgac, 2007). In contrast, when V-ATPase is inhibited, protons accumulate in the cytosol, leading to acid toxicity and cell death. Thus, V-ATPase inhibition in cancer cells may be a catch-22 proposition: loss of V-ATPase activity induces glycolysis for survival in low-oxygen environments, but inactive V-ATPase is incapable of dealing with the consequences of increased glycolysis, thus triggering cell death.

## V-ATpase Integrates Glycolytic Signals to Control Cellular Metabolism

In our final section, we will discuss how many of the disparate metabolic cues underpinning the interplay between V-ATPase and glycolysis converge on a protein super-complex at the surface of lysosomes. This complex includes V-ATPase, mTORC1, and AMPK and reversibly controls the metabolic switch between catabolism and anabolism in response to glucose deprivation and replenishment (Figure 2v).

Under nutrient-rich conditions, the protein kinase mTORC1 phosphorylates a variety of targets to stimulate cell growth and integrate multiple nutrients and energy signals to sustain cellular homeostasis (Efeyan et al., 2012; Jewell et al., 2013). In contrast, during nutrient scarcity, mTORC1 inhibition causes the cells to switch from anabolic to catabolic metabolism and, eventually, a quiescent state. The role of V-ATPase in mTORC1 sensing of amino acids in lysosomes is well-established (Zoncu et al., 2011; Stransky and Forgac, 2015). It is less understood how the V-ATPase-mTORC1 complex responds to glucose signals; however, recent work suggests that it may involve a sequestration of V-ATPase by AMPK (Figure 3B). In the presence of glucose, V-ATPase sits in a complex with Ragulator and interacts with mTORC1. Glucose depletion allows binding of AXIN/LKB1, which phosphorylates and activates AMPK (Zhang et al., 2014, 2017). This process pulls the V-ATPase-Ragulator complex away from mTORC1, shutting down mTORC1 activity and thereby providing a switch between anabolism and catabolism.

Fascinatingly, this interaction seems to involve sensing of the glycolytic intermediate F1,6BP and a functional interaction with the glycolytic enzyme aldolase (Zhang et al., 2017). When aldolase is unoccupied by its F1,6BP substrate, formation of the V-ATPase-Ragulator-AMPK complex is favored (Figure 3B, *left*), and AMPK activity increases as F1,6BP decreases. Adding F1,6BP disrupts the AXIN/LKB1 interaction with V-ATPase-Ragulator, favoring formation of the V-ATPase-Ragulator-mTORC1 complex instead (Figure 3B, *right*). Thus, aldolase appears to serve as both a sensor of glucose and a regulator of AMPK. Notably, upon removal of F1,6BP, AMPK activity will increase even if glucose levels are high, independent of the usual requirement for AMP activation. This process is also independent of aldolase activity, as catalytically inactive mutants that can still bind F1,6BP will block AMPK activation.

Notably, V-ATPase must be inactive for this cascade to happen (Zhang et al., 2013, 2014), and pharmacological inhibition of V-ATPase triggers formation of the AMPK-activating complex even in high glucose conditions. This suggests that V_1_V_o_ disassembly may be a prerequisite for AMPK activation. However, no one has yet established a direct link between V_1_V_o_ reassembly and AMPK inactivation. One hypothesis is that when aldolase is not bound to F1,6BP (low glycolysis), V-ATPase is inactive/semi-dissociated, allowing it to form the AMPK-activation complex. When glycolysis is reinitiated, F1,6BP binds to aldolase to reassemble V-ATPase, and V-ATPase activity turns on the mammalian target of rapamycin complex 1 (mTORC1)-activation complex.

V-ATPase-AMPK regulation functions in both directions. A study showing that acute glucose depletion increased V-ATPase assembly and activity in kidney cell cultures (McGuire and Forgac, 2018; Parra and Hayek, 2018) concluded that AMPK was upregulated first (within 1 min), before V-ATPase activity was elevated (5 min later). These results differ from the above data on the role of V-ATPase in activating AMPK (Zhang et al., 2017) and the discrepancy will likely be an active area of further research.

Although a fungal lysosomal super-complex has not yet been established, a pathway involving many of the same players appears to be present in yeast (Figure 3A, *right*). Active V-ATPase is required for TORC1 activation (Deprez et al., 2018). TORC1 signaling involves activation of EGOC Rag GTPase Gtr1/2 (functionally equivalent to mammalian Ragulator) followed by TORC1 phosphorylation of the protein kinase Sch9 (S6 kinase yeast ortholog) at the vacuolar membrane (Urban et al., 2007; Binda et al., 2010; Panchaud et al., 2013). Sch9 serves as the primary mediator of anabolism during TORC1 activation, and Sch9p interacts with the V_1_ domain of V-ATPase in the presence of glucose (Wilms et al., 2017). Following glucose depletion, Snf1p (AMPK yeast orthologue) phosphorylates the TORC1 components Kog1p (Hughes Hallett et al., 2014, 2015) and Sch9p (Lu et al., 2011). This triggers Sch9p to dissociates from the V_1_ domain of V-ATPase via an unknown mechanism (Wilms et al., 2017), and notably, V-ATPase disassembly is reduced in *sch9Δ* mutants. Taken together, these data suggest that perhaps active Sch9p creates a negative feedback response provoking V-ATPase disassembly and inactivating TORC1 after glucose removal. Although the yeast and mammalian systems differ, many elements of the interaction between V-ATPase, TORC1, AMPK, and glucose are conserved. Tying V-ATPase activity to the metabolic state of the cell seems to be an ancient and successful strategy for energy preservation under nutrient-scarce conditions.

## Conclusion

Cells adjust V-ATPase assembly, activity, and membrane distribution as a reflection of the glucose and energy levels within the cell. The glycolytic enzyme PFK-1 regulates the flux through glycolysis; in yeast cells, this influences V-ATPase proton transport at steady state and reversible disassembly following glucose depletion. Additionally, the Ras/PKA glucose signaling cascade controls reassembly, and V-ATPase may interconnect PFK-1 and PKA glucose pathways. In mammalian cells, glycolysis additionally influences V-ATPase-regulated trafficking through signaling cascade pathways including PI3K and PKA. In mammals, V-ATPase inhibition reciprocally stimulates downstream glycolysis via HIF-1, which steers the cells into a stage that mimics the Warburg effect. Finally, these glucose-sensing pathways appear to converge at the mammalian lysosome, where the F1,6BP glycolytic metabolite allows inactivation of AMPK and activation mTORC1 through a super-complex that includes aldolase, V-ATPase, AMPK, and mTORC1. Thus, understanding how information from glycolysis flows to V-ATPase, and vice versa, allowing cells to fine-tune how much V_1_V_o_ to assemble and/or relocate when glucose is in short supply, can be of great consequences to human health. This is particularly true in cancer therapies, where the mechanisms allowing for metabolic plasticity and adaptation to low glucose environments are increasingly important.

## Author Contributions

KP conceived the idea for this review. KP and SH wrote the manuscript. HSR made the figures. KP, SH, and HSR edited the manuscript.

## Conflict of Interest Statement

The authors declare that the research was conducted in the absence of any commercial or financial relationships that could be construed as a potential conflict of interest.
